# One- or two-step? New insights into two-step hypothesis and rainbow-like theory for pitch class–color synesthesia

**DOI:** 10.3389/fpsyg.2024.1482714

**Published:** 2025-01-10

**Authors:** Ang Cao, Kazuhiro Ueda

**Affiliations:** Graduate School of Arts and Sciences, The University of Tokyo, Tokyo, Japan

**Keywords:** synesthesia, pitch class–color synesthesia, dual task, absolute pitch, rainbow-like theory

## Abstract

**Introduction:**

This study investigates the mechanisms underlying pitch class–color synesthesia, a cognitive trait in which musical pitches evoke color perceptions. Synesthesia in music particularly involves the association of pitch classes (e.g., do, re, and mi) with specific colors. A previous study introduced the two-step hypothesis, which suggests that pitch class identification precedes color association, and proposed a *rainbow-like* theory based on color gradients selected by synesthetes. The primary objective is to retest these theories to evaluate their generalizability in explaining pitch class–color synesthesia.

**Methods:**

We employed a dual-task paradigm to assess the robustness of the two-step hypothesis and conducted qualitative interviews to explore the nature of synesthetic experiences.

**Results:**

The results indicated that the two-step hypothesis may not be always applicable, because it effectively accounts for only a subset of synesthetes. The presence of one-step synesthetes, who experience direct pitch-to-color associations without intermediate steps, implied a more varied synesthetic mechanism. Moreover, rainbow-like theory predominantly characterized two-step synesthetes, while one-step synesthetes exhibited distinct color perceptions. Furthermore, we found that the differentiation between two- and one-step synesthesia may be associated with the methods through which participants develop synesthetic associations.

**Discussion:**

The findings highlighted the diversity of synesthetic experiences in pitch class-color synesthesia, which challenges the generalizability of the current theories and poses the need for a further nuanced understanding of this phenomenon.

## 1 Introduction

Synesthesia is a fascinating perceptual phenomenon characterized by an atypical interweaving of sensory experiences, in which the stimulation of one sensory or cognitive pathway involuntarily triggers an experience in another. This sensory crossover, which can be traced back to the Greek terms “syn” (together) and “aesthesis” (perception), leads to unusual experiences, such as *seeing* sounds as colors or *tasting* words (Cytowic, 2002). Despite the varying prevalence rates, scholars currently estimated that synesthesia occurs in approximately 1%–4% of the population (Simner et al., 2006). The diversity of synesthetic experiences is immense in terms of the types of sensory combinations and the subjective quality of these perceptions. This suggests that synesthesia may not be a single unified condition; instead, it is a spectrum of interrelated phenomena (Novich et al., 2011). In the realm of synesthesia, an important concept that merits further exploration is the so-called *critical period*, i.e., a stage in life during which an organism is particularly sensitive to certain environmental stimuli. Experiences during this period can result in long-term changes to perceptual and cognitive functions (Knudsen, 2004). In the context of synesthesia, research points to the existence of a critical period during which synesthetic associations are formed (Maurer and Mondloch, 2005). This perspective is consistent with observations that the onset of synesthetic experiences frequently occurs during early childhood and remains remarkably consistent across the lifespan (Simner and Logie, 2007).

An intriguing subtype of this phenomenon that intersects with the realm of music cognition is *pitch class–color synesthesia*. This form of synesthesia denotes the consistent association between particular musical pitches or pitch classes and specific colors (Ward et al., 2006). This phenomenon introduces a unique interface between auditory and visual perceptual experiences (estimated to occur in less than 1% of the population; Simner et al., 2006). For individuals with this variant of synesthesia, each musical note or key, such as do, re, mi, or fa (C, D, E, F etc.), consistently induces the perception of a specific color (Sagiv et al., 2006). The underlying mechanisms and implications of this sensory crossover remain topics of active investigation, and understanding them promises to elucidate the complex interplay between the auditory and visual perceptions in the cognitive system.

**FIGURE 1:**
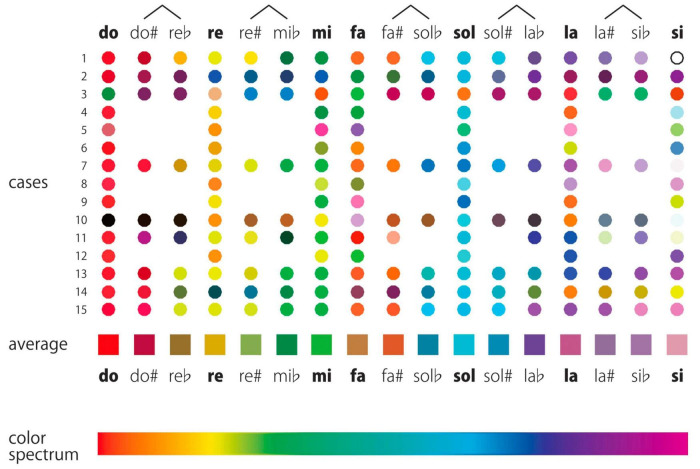
Results of the color-selection test in Itoh et al. (2017). Colors selected by individual participants are shown as circles arranged in rows and the averaged colors are shown with square. Figure reproduced from Figure 2 in Itoh et al. (2017).

**FIGURE 2:**
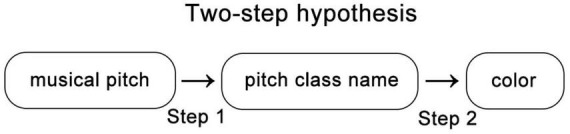
Two-step hypothesis (Itoh et al., 2017). Musical pitches are first identified by their respective pitch class names, and these names are then associated with specific colors. This figure was created by the authors based on Figure 1 in Itoh and Nakada (2018).

The seminal study of Itoh et al. (2017) presented invaluable insights into the realm of pitch class–color synesthesia. This research proposed *rainbow-like* theory and the *two-step* hypothesis for this atypical synesthetic experience. The authors focused on the intricate mappings between pitch classes and color perceptions in pitch class–color synesthetes. Notably, their work presented evidence for the existence of systematic rainbow-like color mappings across pitch classes. In the experiment, the participants with pitch class–color synesthesia selected the colors they perceived from each pitch in a color selection test, and the results indicated that the average color gradient resembled a rainbow (Figure 1). This finding posed significant implications for the study on synesthetic perception and cognition. In this meticulously designed study, the authors also examined the potential cognitive mechanisms that underpin these associations. Itoh et al. (2017) designed several tasks and found that participants generally responded more quickly when identifying colors/pitch names from syllables compared with when identifying them from auditory stimuli. Moreover, the responses were generally faster for reporting pitch names than those for reporting colors. The authors claimed that extended reaction times (RTs) were a result of the experience of the participants of two steps, which, thereby, indicates their *two-step hypothesis* (Figure 2). According to this hypothesis, pitches are first identified through the respective names of their pitch classes; these names are then associated with specific colors. However, the robustness of this hypothesis requires further tests. Given the significant individual variability in synesthetic abilities, a possibility exists that the rainbow-like theory and the two-step hypothesis can only explain a few of the mechanisms that occur in synesthesia possessors. In fact, Itoh et al. (2017) mentioned that two of the participants claimed that their mechanisms of pitch class–color synesthesia were inconsistent with the two-step hypothesis. Further research is necessary to determine the extent to which the hypothesis of Itoh et al. (2017) can be generalized to all synesthetes who exhibit pitch class–color synesthesia. In addition, the majority of the results of the authors were obtained using quantitative methods, which may render explaining the various individual differences difficult. To address these concerns, the current study incorporates additional qualitative analyses based on interviews. This aspect leads to the primary objective of the study: to retest rainbow-like theory and the two-step hypothesis proposed by Itoh et al. (2017) regarding pitch class–color synesthesia and ascertain its scope of applicability.

Absolute pitch (AP) is a rare cognitive trait characterized by the ability to recognize and name the pitch of a musical note without a reference pitch (Ward and Burns, 1982). A few previous studies indicated a potential relationship between AP and synesthesia. For example, Gregersen et al. (2013) found that out of 768 participants with AP, 151 (20.1%) reported synesthesia, which indicates a much higher rate than the 4% reported for the general population. In their study, Loui et al. (2012) mentioned that the AP and synesthesia groups displayed higher levels of activation of the superior temporal gyrus on both sides than those of the control group. Moreover, the developments of AP and synesthesia were reported to occur during a critical period; in other words, they may only be acquired during a certain period of time, i.e., typically at childhood (AP possessors typically report early, preschool, musical training, Russo et al., 2003. Learning of specific sequences during an early critical period may be critical for acquisition of synesthesia, Rich et al., 2005). Among the forms of synesthesia, pitch class–color synesthesia is seemingly particularly associated with AP. Pitch class–color synesthesia and AP involve a unique perception of musical notes. Although in one of their follow-up studies Itoh et al. (2017) empirically demonstrated that possessing pitch class–color synesthesia does not necessarily entail acquiring AP (Itoh and Nakada, 2018), the current study still aims to explore another potential relationship between these two abilities through interviews. Indeed, this background constitutes the secondary purpose of the study.

## 2 Overview of the experiment

As mentioned in the Introduction, the main objective of the current study is to empirically retest rainbow-like theory and the two-step hypothesis posited by Itoh et al. (2017) regarding pitch class–color synesthesia and to elucidate the extent of its generalizability. Additionally, we aim to further explore a potential correlation between AP and pitch class–color synesthesia through interviews.

This study tested the robustness of the two-step hypothesis by Itoh et al. (2017) using a dual-task paradigm. As a critical tool in cognitive psychology and neuroscience, scholars frequently use it to understand the capacity and limits of cognitive processing. It involves concurrent performance in two tasks, which are typically designed to assess the cognitive resources required for each task and to elucidate how they may interfere with each other (Pashler, 1994). The central idea of the dual-task paradigm is that if two tasks are simultaneously performed and if performance deteriorates compared with that when these tasks are performed separately, then they likely share common cognitive resources, which indicates a cognitive bottleneck, and vice versa (Meyer and Kieras, 1997).

Several studies demonstrated that pitch name recognition is an ability related to verbal capacity (Hou et al., 2017). Brown et al. (2006) found the activation of overlapping brain regions during musical and language tasks, which provides neurophysiological evidence for the link between musical and verbal abilities. Burns and Ward (1999) proposed a framework for arguing that the use of pitch in language can be viewed as a form of pitch naming, which further points to a connection between pitch name recognition and verbal capacity. Based on the aforementioned exposition, the present study employs the dual-task paradigm in an experiment to demonstrate that a verbal task interferes with the pitch name recognition process among persons with pitch class–color synesthesia. If the two-step hypothesis holds, then synesthetes would first convert sound into a pitch name after hearing a pitch and subsequently experience color concurrent with the pitch name. Consequently, interference with the first step of pitch recognition in the synesthetes would similarly lead to a disruption in color perception, which indicates that performances on the pitch naming and color correspondence tasks will decrease. If the proposed result is indeed obtained through interference, then it would be evidence of the two-step hypothesis of Itoh et al. (2017).

Based on the aforementioned research policy, the experimental design employed by the study is as follows. It used a 2 (response: color/pitch) * 3 (interference: none/visual/verbal) within-participant factorial design; specifically, the within-participant variables are response and interference.

The reason for including the two response types is that participants were instructed to report the perceived color or the pitch name of the auditory stimuli presented during the experiment. Meanwhile, the reason for including the three interference conditions, namely, no interference (none), interference during a visual judgment task (visual interference), and interference during a verbal judgment task (verbal interference), is that the participants needed to concurrently perform different interference tasks while responding to color or pitch name. As previously explained, if Itoh et al.’s (2017) two-step hypothesis is true, then the participants under the verbal interference condition, which shares common cognitive resources with pitch name recognition, are expected to reduce performance in color and pitch name responses. The reason is that, according to the two-step hypothesis, the participants must undergo a pitch name recognition process (the first of the two-step process) regardless of whether they respond with a color or pitch name.

In addition to six sessions with different combinations of variable levels, this study conducted two additional sessions to test the difficulty of interferences for a total of eight sessions. The following section will describe the details of each session.

Furthermore, this study validates rainbow-like theory through a self-reported questionnaire and investigates the relationship between AP and pitch class–color synesthesia through interviews. The participants will respond to the colors they perceive from various sounds prior to the experiment and a series of inquiries regarding AP perception and synesthetic experience after the experiment.

## 3 Materials and methods

### 3.1 Participants

The study recruited the participants through poster advertisements at the university to which the authors belong and via personal introduction. They received 2,000 yen (approximately 15 USD at the time) as gratuity. The criteria for participation were as follows: normal adult, those who can identify the pitch name of a sound after listening (i.e., possess AP), those who can vaguely imagine a color from a pitch (i.e., possess pitch class–color synesthesia), those with normal or corrected-to-normal vision, and those with normal hearing.

Notably, Itoh and Nakada (2018) asserted that the possession of pitch class–color synesthesia does not necessarily entail the simultaneous presence of AP with which the current study concurs. However, given that the current study aims to further explore the relationship between AP and pitch class–color synesthesia through interviews, participants with self-reported AP were recruited.

Eighteen participants who met the abovementioned criteria through self-report registered for the experiment. Table 1 provides specific information on the participants.

**TABLE 1:** Information of the participants (*n* = 18).

Participant	Age	Gender	Most difficult session (Q1)	Mechanism (Q2)	Age to start music training (Q3)	Age to acquire synesthesia (Q4)	Age to acquire AP (Q5)
**P1**	**40s**	**f**					
**P2**	**40s**	**f**	**Color+Visual**	**Two-step**	**0–5**	**6–10**	**6–10**
**P3**	**40s**	**f**		**One-step: dual tasks, two-step: solo tasks**			
**P4**	**20s**	**m**	**Color+Visual**	**Two-step**	**15–20**	**15–20**	**0–5**
**P5**	**20s**	**m**	**Pitch+Visual**	**Two-step**	**0–5**	**0–5**	**0–5**
**P6**	**10s**	**f**	**Color+Visual**	**One-step**	**0–5**	**6–10**	**6–10**
**P7**	**20s**	**f**	**Pitch+Verbal**	**Two-step**	**0–5**		
**P8**	**20s**	**f**	**Pitch+Verbal**	**One-step for do∼la, two-step for si**	**15–20**	**15–20**	**6–10**
**P9**	**20s**	**f**	**Pitch+Verbal**		**0–5**	**6–10**	**0–5**
P10	20s	f	Pitch+Visual	Two-step	**0–5**	**0–10**	**0–10**
P11	10s	m	Color+Visual	Two-step	**0–5**	**6–10**	**6–10**
P12	10s	f	Color+Verbal	Two-step	**0-5**		
P13	10s	f	Color+Visual	One-step	**0–5**	**0–5**	**0–5**
P14	10s	f	sessions with Pitch	Two-step	**0–5**		
P15	10s	m	Pitch+Verbal	Two-step	**0–5**	**10–15**	**6–10**
P16	10s	f	Color+Visual	Two-step	**6–10**		**6–10**
P17	10s	m	/	/	**/**	**/**	**/**
P18	10s	m	/	/	/	/	/

In the gender column, f means female and m means male. A blank field indicates that the participant either did not respond or was unsure of the answer to the question. Data from P17 and P18 were excluded because they did not meet the criteria (detailed in section “4.1 Dataset and methods”).

### 3.2 Experimental environment and equipment

The experiment was conducted in a quiet room of the university to which the authors belong. The heights of the desk and chair were adjusted to the most comfortable state dependent on the participants prior to the experiment.

Data collection was conducted using a MacBook Air (2020, 13-inch) laptop and a wired mouse; the brightness of the computer screen was consistently maintained across the participants.

### 3.3 Stimuli

The auditory stimuli used in the experiment consisted of 14 2-second piano tones (i.e., 3G, 3A, 3B, 4C, 4D, 4E, 4F, 4G, 4A, 4B, 5C, 5D, 5E, and 5F), and 1-second white noise. Piano tones and white noise were generated using Overtone 5 and Adobe Audition 2020, respectively. The volume of all generated materials was adjusted, such that the sound level remained constant using Adobe Audition 2020.

The textual stimuli were 56 Japanese words, each composed of two Kanji characters. All words consisted of two syllables with the number of strokes for each character ranging from 12 to 18. Based on pronunciation and glyph, the words were categorized into the four types (see Table 2 for specific textual stimuli):

1. words with /N/ in their pronunciation and have a closed area surrounded by lines in their glyph such as “
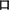
” (closed area+/N/);
2. those with /N/ in their pronunciation but without a closed area in their glyph (only /N/);
3. those without /N/ in their pronunciation but exhibits a closed area in their glyph (only closed area); and
4. those without /N/ in their pronunciation and do not exhibit a closed area in their glyph (no closed area nor /N/).

**TABLE 2:** Four types of textual stimuli.

Closed area + /N/	Only /N/	Only closed area	Neither closed area nor /N/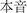 (real intention)	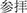 (visit to a shrine)	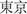 (Tokyo)	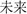 (future)
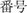 (number)	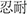 (patience)	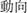 (trends)	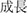 (growth)
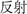 (reflection)	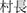 (village chief)	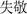 (discourtesy)	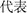 (representative)
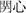 (interest)	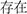 (existence)	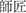 (master)	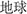 (earth)
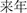 (next year)	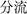 (bifurcation)	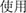 (use)	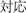 (correspondence)
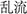 (turbulence)	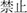 (prohibition)	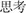 (thought)	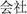 (company)
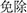 (exemption)	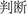 (judgment)	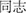 (comrade)	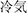 (cold air)
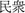 (people)	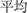 (average)	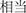 (equivalent)	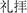 (worship)
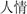 (human empathy)	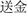 (remittance)	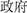 (government)	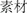 (material)
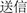 (transmission)	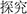 (inquiry)	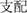 (domination)	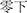 (below zero)
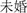 (unmarried)	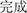 (completion)	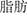 (fat)	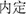 (job offer)
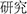 (research)	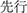 (preceding)	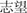 (aspiration)	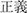 (justice)
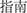 (guidance)	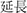 (extension)	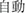 (automatic)	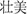 (magnificence)
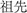 (ancestor)	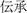 (tradition)	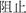 (prevention)	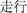 (running)

Through this design, the same word can function as a visual and a verbal interference. As a visual interference, the participants judged whether or not stimuli contain a closed area; as a verbal interference, the participants identified the presence of the phoneme /N/ in the pronunciation of stimuli.

The font style of the textual stimuli was uniformly set to STSong. In each session, the same stimulus appeared only once, and the order of appearance was randomized for each participant.

### 3.4 Procedure

#### 3.4.1 Questionnaire

Prior to the experiment, the participants received an online questionnaire via email, which asked them to answer the code numbers of the colors that they could perceive from the seven pitches: do, re, mi, fa, so, la, and si (In Japan, where the data were collected, this representation is more widely recognized than C, D, E, F etc.). We did not conduct a rigorous consistency as provided by Itoh et al. (2017). For details on the specific impact of this, please refer to section 1.3 of the Supplementary material.

#### 3.4.2 Behavioral experiment

The study conducted eight experimental sessions per participant. These sessions consisted of four types of solo tasks (i.e., color, pitch, visual, and verbal) that were conducted individually or in pairs (Table 3). Prior to each session, the participants were given the opportunity to practice to familiarize themselves with the experimental operations. The practice could be repeated as desired. After every three sessions, the participants took a 2-min break until all eight sessions were completed.

**TABLE 3:** Combination of tasks across sessions.

Interference Response (Main task)	Visual	Verbal	None
Pitch	Session 7	Session 8	Session 2
Color	Session 5	Session 6	Session 1
None	Session 3	Session 4	

**FIGURE 3:**
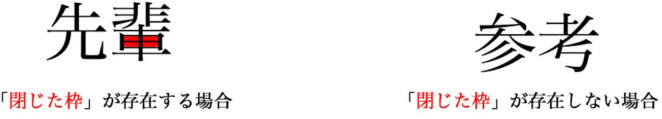
The word on the left is a word with a closed area, while that on the right is a word without a closed area.

Among the four abovementioned tasks, the study analyzed the color and pitch (target) tasks, while visual and verbal tasks were the interference tasks. For each session, one target task was combined with one interference task. Participants were instructed to respond to both tasks simultaneously, ensuring equal focus on both tasks. We analyzed whether the performance of the participants in the target task changed under the influence of the interference tasks. The details of the four solo tasks were as follows: (1) color: listen to a piano tone and identify with the color feeling based on the tone; (2) pitch: listen to a piano tone and determine the pitch name of the tone; (3) visual: look at a word and determine the shape of the word (in this case, the presence of a closed area; for example, see Figure 3); and (4) verbal: look at a word and judge whether or not the word contains /N/ in the pronunciation.

The order of the eight sessions was counterbalanced among the participants using a Latin square design. Table 4 outlines the specific task descriptions for the experiment.

**TABLE 4:** Description of each session.

Session	Display	Task
1	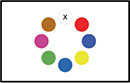	[Color] Use the mouse to click the color you perceive from the piano tone as soon as possible.
2	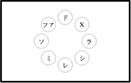	[Pitch] Use the mouse to click the syllable you perceive from the piano tone as soon as possible.
3	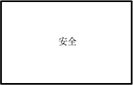	[Visual] Use space key to judge whether or not a closed area exists in the shown word. If there is a closed area, then please press the space key as soon as possible.
4	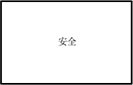	[Verbal] Use the space key to determine whether or not an “/N/” pronunciation exists in the shown word. If there is an “/N/”, then please press the space key as soon as possible.
5	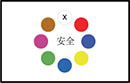	[Color + Visual] Use the mouse to click the color you perceive from the piano tone and simultaneously press the space key to judge whether or not a closed area exists in the shown word as soon as possible.
6	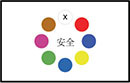	[Color + Verbal] Use the mouse to click the color you perceive from the piano tone and simultaneously press the space key to identify whether or not an “/N/” pronunciation exists in the shown word as soon as possible.
7	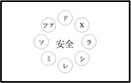	[Pitch + Visual] Use the mouse to click the syllable you perceive from the piano tone and simultaneously press the space key to determine whether or not a closed area exists in the shown word as soon as possible.
8	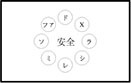	[Pitch + Verbal] Use the mouse to click the syllable you perceive from the piano tone and simultaneously press the space key to identify whether or not an “/N/” pronunciation exists in the shown word as soon as possible.

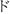
, 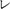, 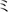, 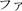, 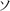, 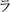, 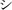 are the Japanese transliterations of do, re, mi, fa, so, la, and si. The term “
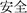
” means “safe” in Japanese. It is used as an example of a textual stimulus.

**FIGURE 4:**
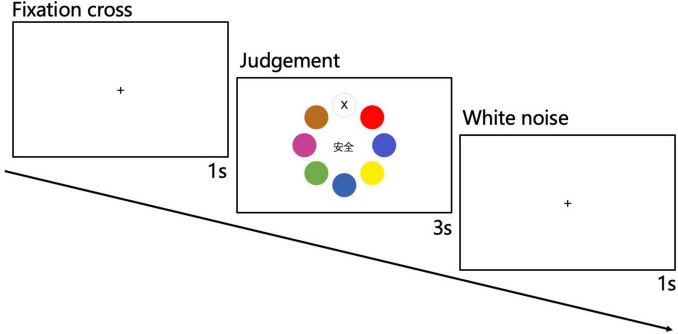
Example of a single trial. The participants first saw the “+” symbol for 1 s, then made judgment within 3 s, and, finally, heard a 1-s white noise.

For each session, each piano tone explained in section “3.3 Stimuli” was presented four times in an entirely random sequence. Thus, each session consisted of 56 trials. For each trial, a fixation cross was displayed at the center of the screen. One second after the fixation, textual stimulus and options appeared on the screen concurrent with the presentation of a 2-second piano tone. The participants were required to respond within 3 s using the mouse and spacebar. After the judgment, the screen will go blank accompanied by a 1-second white noise regardless of whether or not the participants provided a response (Figure 4).

#### 3.4.3 Post-experimental interview

Finally, the participants were interviewed to gain insights into the development and nature of their synesthetic/AP experiences. The specific questions asked in the interviews were as follows:

Q(1) Which of the eight sessions do you find the most difficult?
Q(2) Do you believe that your synesthesia is a pitch–pitch name–color process (i.e., the process of recognizing the name of a note from its pitch and perceiving the color from its name, i.e., the two-step process proposed by Itoh et al., 2017), a pitch–color process (i.e., the process of perceiving color directly from the pitch), or other processes?
Q(3) What was your music learning experience and duration of music training?
Q(4) When and how did you acquire synesthesia?
Q(5) When and how did you acquire AP?
Q(6) Do you have any other forms of synesthesia apart from pitch class–color synesthesia (regardless of the process, one can feel color directly from pitch)?
Q(7) Are there any other characteristics unique to your synesthesia (for example, is it affected by different sounds/context/moods)?

### 3.5 Data collection and analysis

This study used PsychoPy for data collection and MATLAB, SPSS Statistics 26, and Excel for data analysis. The RT of the key presses and mouse selections of the participants were recorded.

### 3.6 Expected results (hypotheses)

The study proposes a total of four expected results as follows:

H1 According to the results of Itoh et al. (2017), if the two-step hypothesis is valid, the RT for color task should be longer than for pitch task.

H2 The same task performed under the dual-task or interference conditions (Session 5/6/7/8) would exhibit poorer performance due to the increased cognitive resource demand than that under the solo conditions (Session 1/2).

H3 According to Itoh et al. (2017), participants’ performance both on the pitch task and on the color task will be significantly worse in verbal interference sessions (Session 6/8) than in visual interference sessions (Session 5/7).

H4 The averaged self-reported colors in the color selection questionnaire will exhibit a rainbow-like pattern.

## 4 Results

### 4.1 Dataset and methods

After a thorough examination, the results indicated that two participants (P17 and P18) did not meet the experimental requirements. In the pitch naming task, P17 achieved a correct response rate of only 28.6%, which indicates that he does not possess AP. In the post-experiment interview, he mentioned that he could only accurately identify *do*, which implies that he may possess partial AP. Similarly, P18 achieved a correct response rate of only 28.6% in the color corresponding task and admitted in the post-experiment interview that he may not have synesthesia. Thus, their data were excluded. Thus, for subsequent analysis, data from 16 participants were used.

To test the hypotheses in section “3.6 Expected results (hypotheses),” we first analyzed RTs (as the benchmark of performance mentioned in the hypotheses) across sessions (see section “4.2 Analysis of RTs”). The RTs were analyzed using two-way repeated-measure ANOVA; specifically, one factor was assigned as a response (two levels: pitch/color) while the other was denoted as interference (three levels: none/visual/verbal). We then analyzed the colors perceived from different pitches, which the 16 participants filled out in the online questionnaire, and averaged these colors to compare with the result of Itoh et al. (2017; section “4.3 Interview”). We then performed a qualitative analysis of the post-experimental interview content (section “4.4 Supplementary analysis based on the interview results”). Finally, we reanalyzed the experimental data in conjunction with the content of the interviews.

### 4.2 Analysis of RTs

#### 4.2.1 Verification of H1, H2, and H3

To test H1, we compared RTs between the pitch and color tasks. Based on the results of Itoh et al. (2017), we expected that the current participants would spend more time on color tasks than they would on pitch tasks (H1). The results of the two-way repeated-measure ANOVA with response (two levels: pitch/color) and interference (three levels: none/visual/verbal) as the dependent variables yielded a nonsignificant main effect for response (*F* = 0.400, *p* = 0.527, η^2^ < 0.001), a significant main effect for interference (*F* = 242.056, *p* < 0.001, η^2^ = 0.213), and a nonsignificant interaction of response * interference (*F* = 1.107, *p* = 0. 331, η^2^ = 0.001). The study found no significant difference in RTs between the two tasks; thus, H1 was not supported, and we were unable to replicate the result of Itoh et al. (2017).

**FIGURE 5:**
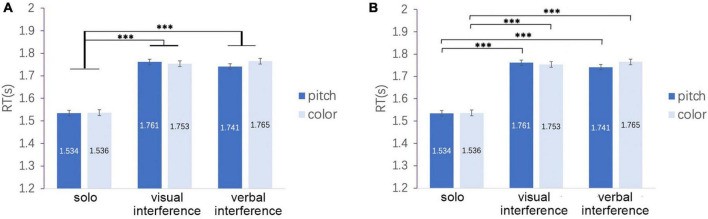
**(A)** RTs under the solo condition were significantly shorter than each of those under the two dual-task conditions, but the study found no significant difference between the visual and verbal interference conditions. **(B)** Result of paired comparisons. Regardless of the type of response, the study found no difference between the visual and verbal interference conditions. In both panels, error bars indicate standard errors. ***p < 0.001.

To test H2 and H3, in addition, we compared RTs under the interference conditions. The result of *post hoc* comparison using Bonferroni’s correction for the abovementioned ANOVA, RTs under the solo task condition was significantly shorter than each of those under the two dual-task conditions (*p* < 0.001 for solo versus visual and for solo versus verbal), which is consistent with H2. However, the study found no significant difference between the visual and verbal interference conditions (*p* = 1.000; Figure 5A), which is inconsistent with H3. Paired comparisons yielded similar results. Regardless of the type of response, the dual-task conditions exhibited longer RTs than those of the solo task conditions (*p* < 0.001), but no difference between the visual and verbal interference conditions was found (Figure 5B).

#### 4.2.2 Summary of analysis results of RTs

On the basis of the abovementioned analysis of RTs, the study concluded as follows for the hypotheses. Inconsistent with H1, the study did not find that the participants exhibited longer RTs in the pitch task than they did in the color task. Consistent with H2, the analysis of RTs demonstrated that the participants displayed worse performance in the dual-task conditions than they did in the solo task conditions. Inconsistent with H3, the study detected no worse performance in the verbal task interference condition than that in the visual interference condition.

### 4.2.3 Verification of H4

**FIGURE 6:**
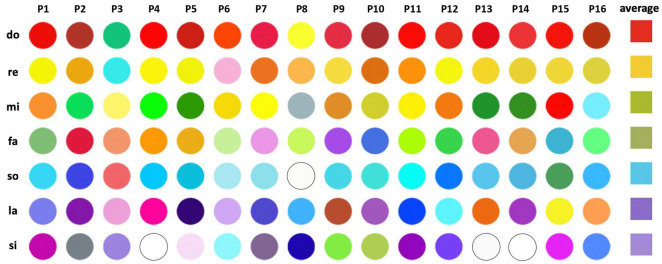
Colors selected by each participant are shown as circles, and the averaged colors are depicted with squares. The last column illustrates the average results of the color selection test.

To test H4, we first analyzed the colors perceived from different pitches, which the participants filled out in the online questionnaire, averaged these colors, and compared with the result of Itoh et al. (2017). Figure 6 presents the specific colors associated with the pitches of do, re, mi, fa, so, la, and si, as selected by the participants. To average colors, we first converted the hexadecimal color code provided by the participants to an RGB color representation that consists of three natural numbers from 0 to 255. We then averaged the R, G, and B numbers of the colors selected for the same pitch by the participants to obtain the average colors. Although we did not perform a statistical test, we found that the color transition from *do* to *si* is approximately red to purple.

**FIGURE 7:**
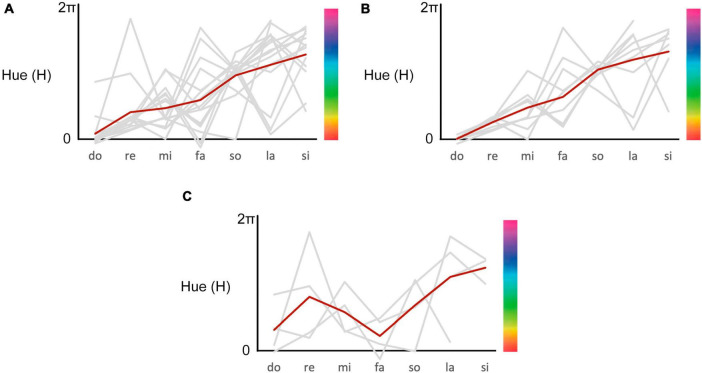
Numerical representation of the colors in Figure 6, plotted for hue value of color in the HSV space. Each gray line represents an individual case, and the red line represents the average hue. **(A)** Colors for all sixteen participants. **(B)** Colors for two-step group. **(C)** Colors for one-step group.

Additionally, we converted the colors selected by the sixteen participants into HSV values (Figure 7A) and conducted a linear regression analysis following the method of Itoh et al. (2017). This regression included random intercepts and slopes for each participant, with a compound symmetry covariance matrix. The dependent variable was the Hue value corresponding to the colors associated from do to si, while the repeated-measures random-effect variable was pitch class (0.00, 0.90, 1.80, 2.70, 3.59, 4.49, and 5.39 radians, corresponding to do through si). The analysis revealed an estimated slope of 0.692 (95% confidence interval [CI]: 0.534–0.850), *t* (107) = 8.543, *p* < 0.001), indicating a significant linear relationship between pitch class and Hue values.

These results are consistent with H4 and align with the findings of Itoh et al. (2017).

### 4.3 Interview

In the interview, we intended to examine the current understanding of the mechanisms of and connections between pitch class–color synesthesia and AP ability by exploring the perceptions of the participants about the behavioral experiment as well as their understanding and formation of their synesthesia/AP.

#### 4.3.1 The most difficult session

Excluding the two participants who were unable to respond to Q1, 14 participants reported the session that they felt was the most difficult.

Among the eight sessions, approximately half of the participants (*n* = 6) considered Session 5 (color + visual) the most difficult. The reasons included “There are no everyday scenarios requiring judgment of closed areas in words, so I find this task rather unfamiliar,” “Color corresponding is less commonly used compared to pitch naming,” and “Judging both color and shape simultaneously feels very difficult.” As hypothesized, only one participant considered Session 6 (color + verbal) the most challenging for two reasons: the first is that color judgment was more challenging than pitch naming, and the second was that color judgment first required pitch naming, and this process conflicted with the silent reading process, which made this dual-task increasingly difficult.

The discrepancy between the perceived difficulty of the task by the participants and the expectations of the researchers is seemingly one of the reasons why H3 was unsupported.

#### 4.3.2 Two- or one-step?

For Q2, 10 out of 14 participants (excluding two participants who did not respond) stated that their process of perceiving color from pitch was two-step, i.e., first discerning the pitch name from the pitch then associating the pitch name with a color sensation, as advocated by Itoh et al. (2017). Many of them mentioned that they could perform pitch naming more quickly than they could color perception.

Out of the remaining four participants, one (P3) stated that she used a one-step cognitive process in the majority of the resource-limited dual tasks, that is, she directly perceived color from pitch without the intermediary of pitch names. During the interview, P3 mentioned that when she directly perceived the color of a pitch, the surrounding context of other pitches influenced the perceived color. As a result, the colors she experienced may differ from those indicated in the pre-experiment questionnaire. This discrepancy ultimately contributed to the lower accuracy (only 57.14%) she obtained in the color corresponding tasks under the dual-task conditions. However, in the solo task, she adopted a two-step process. She said that, in this case, it was more similar to *remembering* instead of *feeling*. Corroborating her statement, she nearly completely reversed the colors corresponding to mi and fa because of miss remembering (only an accuracy rate of 25% in trials in which *mi* and *fa* were presented as stimuli).

Another participant (P8) stated that she used a one-step process to perceive color for the majority of pitches (do, re, mi, fa, so, la) by directly perceiving the colors from the pitches, whereas she adopted a two-step process in color perception only for *si*.

The last two participants (P6 and P13) stated that their color perception for all pitches was a one-step process that did undergo the pitch naming process. P13 specifically mentioned that although she perceived the process of pitch naming was quicker (we analyzed her performance in the pitch and color tasks and found that although statistically nonsignificant, she, indeed, exhibited shorter RTs in the pitch task: 1.569s versus 1.605s in the color task). However, this result does not imply that the more time-consuming color corresponding task needed to be based on pitch naming. For her, the two tasks (pitch naming and color corresponding) are independent processes.

In summary, although the majority of the participants reported that their synesthesia was consistent with the two-step hypothesis of Itoh et al. (2017), several participants reported a distinctive one-step mechanism. This finding may indicate the potential existence of two distinct groups among pitch class–color synesthetes, which is characterized by one- or two-step synesthetic processes. This aspect could also be the potential reason for the failure of the current study to replicate the results of Itoh et al. (2017) regarding H1.

#### 4.3.3 Musical training status of participants

Given that previous studies have demonstrated the crucial role of musical training in the formation of AP, we, following Itoh et al. (2017), gathered information on the participants’ musical training.

The participants provided information regarding their musical training status in response to Q3. All of the participants answered Q3 had a background in musical training. The majority began formal music education prior to school age. However, two participants began learning music later. P4 indicated that although there was a piano at home from his childhood, he never formally learned until he joined choir in college and began systematic music learning. Similarly, P8 mentioned that despite enjoying classical music since junior high school, she only began formal music training after joining the orchestra in university.

#### 4.3.4 Acquisition of AP and synesthesia

The participants provided information regarding their acquisition of AP and synesthesia in response to Q4 and Q5. Except for P4 and P8, all participants reported that they obtained AP during early childhood. P4 stated that he gradually acquired AP through 3 years of choir training in college, and P8 expressed that a formal awakening of AP occurred during university orchestra club activities, although she was *more sensitive* to do, fa, and si due to her fondness for classical music in junior high school. All participants claimed to have acquired synesthesia during early childhood.

Eleven participants mentioned the triggers for their acquisition of synesthesia. These triggers can be roughly divided into two major categories, namely, one-to-one correspondence between sound and single tone and generalization of other sensations.

The first type of acquisition includes instances, such as different notes being marked by different colors on the music score during childhood music training, or the placement of differently colored stickers on piano keys. One person who fell into the second type of acquisition said, “Because a certain piece in C major sounded very inclusive and warm, it felt that the piece is red, and this color sensation generalized from the piece in C major to the single tone C.” However, the second type is not limited to this example.

We conducted further analysis of the self-reports of 10 participants who provided complete responses to Q2 and Q4. Interestingly, we found that the majority (6 out of 7) who self-reported acquired synesthesia through the first type of method reported using a two-step process for color perception. Meanwhile, all of the participants mentioned experiences of generalization in the discussion of their acquisition of synesthesia using a one-step process (3 out of 3) for color perception. One special case is P13, who stated that she acquired synesthesia during ear training during childhood when a teacher asked her to raise a flag corresponding to a specific pitch among flags of different colors. As this training was intended for children who could not read music scores, no pitch names were marked on these flags. Thus, although P13 acquired synesthesia through a one-to-one method, her synesthesia continues to directly link sound to color without the intermediary of pitch names.

#### 4.3.5 Other types of synesthesia

A total of eight participants reported possessing other types of synesthesia apart from pitch class–color synesthesia, the majority of which are also related to music. For example, P2, P3, P6, P8, P9, and P12 reported synesthesia that associates music pieces or chords with specific sceneries, shapes, or temperatures. Meanwhile, P13 and P16 reported synesthetic experiences such as radical–color, number–color, time–color, and month–week–color.

#### 4.3.6 Other characteristics of pitch name–color synesthesia

The participants reported several intriguing characteristics during the interviews. For instance, P2 and P3 noted that the type of musical instrument being played could influence color perception. They also stated that emotional state influenced their synesthetic experiences, i.e., they perceived color according to their mood, and the intensity of color perception changes over time. As if to confirm this, P2 was the only participant who made modifications to the colors she reported in the pre-experimental questionnaire, which may be due to differences in her color perception between the times of filling out the questionnaire and the laboratory experiment. Additionally, P16 pointed out that the same pitch can evoke different color sensations dependent on the musical context. For example, the pitch *do* appeared red in a *do*–*la* chord but changed to blue in a *do*–*mi*–*so* chord. These findings further highlighted the complex and subjective nature of synesthetic experiences and imply that emotional state, musical instrument, and musical context can exert significant effects on these phenomena.

#### 4.3.7 Summary of the interviews

On the basis of the interviews, we obtained the following information. First, for the majority of the participants, the visual + color session was the most difficult one, which may be due to the fact that this session was typically the first dual-task session in the order, and both were unfamiliar tasks that were rarely seen in daily life. Second, although the majority of the participants reported that their synesthesia was consistent with the two-step hypothesis of Itoh et al. (2017), others reported a distinctive one-step mechanism. Third, all participants acquired synesthesia at an early age, while, for AP, two participants indicated that they acquired it after entering college. One of them suggested the facilitative effect of pitch class–color synesthesia on the formation of AP in adulthood. Lastly, a few of the participants reported having other types of synesthesia in addition to pitch class–color synesthesia. Others reported that context, mood, and other factors influenced color perception in their pitch class–color synesthesia.

### 4.4 Supplementary analysis based on the interview results

The results of the interview suggest the possibility of the existence of the two- and one-step processes. Thus, the data of the participants were categorized under the self-reported two-step and self-reported one-step groups (hereafter two- and one-step groups, respectively), and two-way repeated-measure ANOVA was conducted for the individual groups with response (two levels: pitch/color) and interference (three levels: none/visual/verbal) as the independent variables and reaction time (RT) as the dependent variable.

#### 4.4.1 Analysis for the two-step group

This dataset includes all the solo task trials of P3 as well as all trials of P2, P4, P5, P7, P10, P11, P12, P14, P15, and P16.

The results of ANOVA yielded a significant main effect for response (*F* = 11.136, *p* = 0.001***, η^2^ = 0.016), a significant main effect for interference (*F* = 180.123, *p* < 0.001, η^2^ = 0.210), and a nonsignificant interaction of response * interference (*F* = 0.137, *p* = 0.872, η^2^ < 0.001).

**FIGURE 8:**
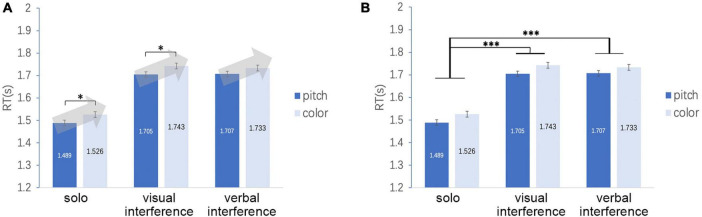
**(A)** RTs for the two-step group. A significant main effect was found for response. Paired comparison indicates that the color task took significantly more time than the pitch task, and the difference in the average RTs between the pitch and color tasks is greater for the participants in the two-step groups than for the 16 participants under the solo and visual interference conditions. **(B)** RTs for the two-step group. A significant main effect was found for interference. RTs under the solo task condition were significantly shorter than each of those under the two dual-task conditions. *p < 0.05, ***p < 0.001.

According to the *post hoc* comparison using Bonferroni’s correction, RTs under the solo task condition was significantly shorter than each of those under the two dual task conditions (*p* < 0.001 for solo versus visual and for solo versus verbal). The paired comparison demonstrated that under the solo task and the visual interference conditions, the color task took significantly more time than the pitch task (*p* = 0.034* for the visual interference condition; *p* = 0.032* for the verbal interference condition; Figure 8A). Although nonsignificant (*p* = 0.135*), the difference in average RTs between the pitch and color tasks under the verbal interference condition (1.733 s–1.707 s = 0.026 s) was greater for the participants in the two-step group compared with all participants (*n* = 16; 1.765 s–1.741 s = 0.024 s). This result is consistent with H1; in other words, the result of Itoh et al. (2017) was replicated in the two-step group. This finding indicates that the two-step hypothesis may only be applicable to a part of individuals with pitch class–color synesthesia.

The abovementioned result, that is, a significant main effect for interference, is also consistent with H2. However, the study found no significant difference between the visual and verbal interference conditions (*p* = 1.000), which is inconsistent with H3 (Figure 8B).

**FIGURE 9:**
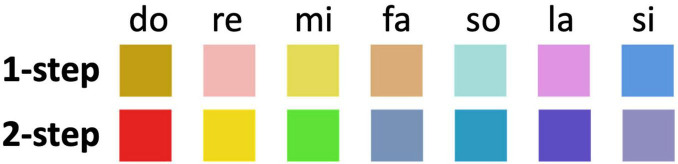
The top panel illustrates the average colors for the one-step group, while the bottom depicts those for the two-step group.

For those who claimed that their color perception for all pitches is a two-step process (i.e., P2, P4, P5, P7, P10, P11, P12, P14, P15, and P16), Figure 9 presents the average color as indicated in the pre-experiment questionnaire (section “4.3 Interview” outlines the method for averaging). Thus, the study infers that the pattern of the average color is consistent with rainbow-like theory.

#### 4.4.2 Analysis for the one-step group

This dataset includes all dual task trials of P3, of P6 and P8 (except for the trials in which the sound stimulus *si* was presented), and of P13.

The ANOVA results yielded a nonsignificant main effect for response (*F* = 3.604, *p* = 0.059, η^2^ = 0.013); a significant main effect for interference (*F* = 151.659, *p* < 0.001, η^2^ = 0.359), and a significant interaction of response * interference (*F* = 4.547, *p* = 0.011*, η^2^ = 0.017).

**FIGURE 10:**
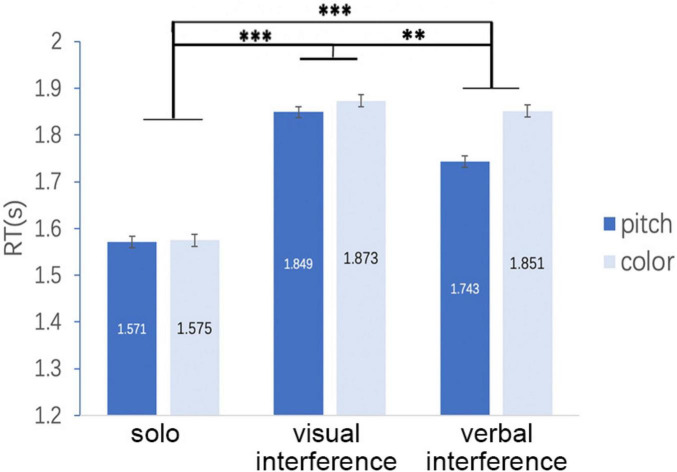
RTs for the one-step group. No difference was found between the color and pitch tasks. RT under the solo task condition was significantly shorter than each of those under the two dual-task conditions (visual and verbal interference conditions). RTs under the verbal interference condition were significantly shorter than those under the visual interference condition. **p < 0.01, ***p < 0.001.

According to the *post hoc* comparison using Bonferroni’s correction, RTs under the solo task condition were significantly shorter than each of those under the two dual task conditions (*p* < 0.001 for solo versus visual and solo versus verbal). RTs under the verbal task interference condition were also significantly shorter than those under the visual interference condition (*p* = 0.002), which does not support H3 (Figure 10).

Although these results may be due to the small sample size (only four) and due to the fact that the order of the sessions among them were not well balanced, a possibility remains that the two- and one-step groups, which exhibited different results, pose extremely different cognitive mechanisms.

Figure 9 presents the average color as indicated in the pre-experiment questionnaire for those who claimed that their color perception for pitch contains a one-step process (P3, P6, P8, I.K; section “4.3 Interview” explains the method for obtaining the average). The study infers that the pattern of the average color is inconsistent with the rainbow-like theory.

#### 4.4.3 Regression analysis for two-step group and one-step group

We conducted the same regression analysis for the two-step and one-step groups as described in section “4.3 Interview”.

For the two-step group (Figure 7B, the results showed an estimated slope of 0.801 (95% confidence interval [CI]: 0.649–0.954), *t* (60) = 10.269, *p* < 0.001), indicating a significant linear relationship between pitch class and Hue values.

In contrast, for the one-step group (Figure 7C), the estimated slope was 0.449 (95% confidence interval [CI]: −1.418 to 2.315), *t* (23) = 0.472, *p* = 0.637), indicating a non-significant linear relationship between pitch class and Hue values.

Although the regression data for the one-step group have limited interpretive value due to the small sample size, the results suggest that the two-step group’s regression is more closely aligned with the hue-pitch class relationship than the findings from the full set of 16 participants, indicating a closer correspondence to the rainbow-like pattern. By contrast, the one-step group did not exhibit a significant relationship.

#### 4.4.4 Performance of two-step group and one-step group in the color task

We did not expect participants in the one-step group to have faster reaction times (RTs) in the color task compared to the two-step group. In fact, the RTs for the two groups were very similar: the mean RT for the two-step group was approximately 1.526 seconds, while that for the one-step group was 1.575 seconds, with the one-step group even showing slightly longer RTs.

We propose that although the one-step group may directly perceive color from pitch, this does not necessarily imply a faster process. The “one-step” mechanism does not inherently guarantee greater speed compared to the “two-step” mechanism. It is likely that the cognitive processes underlying the one-step and two-step mechanisms differ fundamentally, making it unreasonable to infer RT differences solely based on the number of steps involved.

#### 4.4.5 Summary of the supplementary analysis

We divided the participants into two- and one-step groups and analyzed the data for individual groups. As a result, we obtained the following results. For the two-step group, RTs for the color task were significantly longer than those for the pitch task, which is consistent with H1. Alternatively, for the one-step group, the study found no significant difference in RTs between the pitch and color tasks, which is inconsistent with H3. This finding indicates that the mechanism of pitch–color synesthesia for only a portion of the participants were consistent with the hypothesis of Itoh et al. (2017). By averaging the color selected by the participants separately for the two- and one-step groups, the study infers that the color pattern for only the two-step group is consistent with rainbow-like theory, while that for the one-step group is relatively inconsistent.

## 5 General discussion

### 5.1 Summary of results

This study, which aimed to test the hypothesis presented in section “3.6 Expected results (hypotheses),” used a multifaceted approach that involves quantitative and qualitative analyses. Initially, we focused on the perceived colors associated with different pitches as reported by the 16 participants in an online questionnaire and averaged the colors to determine whether or not they were similar to the findings of Itoh et al. (2017). Afterward, the study conducted analysis of the RTs of the participants across sessions using two-way repeated-measure ANOVA. The dependent variables were the types of response (pitch/color) and of interference (none/visual/verbal). Moreover, it performed a qualitative analysis of the post-experimental interviews, which was then integrated with the reanalysis of the experimental data.

The result indicated that the average of colors selected by the participants exhibited a rainbow-like pattern, which resonates with the findings of Itoh et al. (2017). However, the current analysis of RTs led to several unexpected findings. Contrary to the initial hypothesis, the pitch task did not result in longer RTs compared with those of the color task. Consistent with another hypothesis, we observed worse performance in the dual-task conditions than that in the solo conditions but found no significant difference in performance between the verbal and visual interference conditions, which challenges the initial hypotheses.

The interviews offered further insights. Although the majority of the participants reported that their synesthesia was consistent with the two-step hypothesis of Itoh et al. (2017), several participants reported a distinctive one-step process. Interestingly, all participants acquired synesthesia at an early age. For AP, however, two participants indicated that they acquired it after entering college. One of them suggested a facilitative effect of pitch class–color synesthesia on the formation of AP in adulthood. Additionally, a few participants reported their experience with other types of synesthesia and noted that various factors, such as context and mood, influenced their color perceptions.

The revelations about the potential existence of two- and one-step synesthetic processes during the interviews led the researchers to categorize the participants accordingly. In the two-step group, the RTs for the color task were significantly longer than those for the pitch task, which aligns with H1. In the one-step group, however, the study found no significant difference in RTs between the pitch and color tasks. These results indicate that synesthetic mechanisms could vary among individuals. Moreover, the study analyzed the color selections for both groups and found that color pattern of only the two-step group matches rainbow-like theory, whereas that of the one-step group does not.

### 5.2 Existence of the one-step mechanism apart from the two-step mechanism

The primary objective of this study was to retest the conclusions drawn by Itoh et al. (2017). However, empirical data in this study of the current study did not corroborate the assertion of the authors that the cognitive process of pitch class–color synesthesia is two-step, that is, synesthetes first perceive the pitch name from the pitch then feel the color from the pitch name.

Comparing the RT performance of the participants in the color and pitch tasks regardless of interference conditions, the results did not replicate those of Itoh et al. (2017), i.e., the study found no significant differences in RTs between the two tasks. Moreover, the comparison of performances in the color and pitch tasks under the different interference conditions revealed results that are inconsistent with H3.

Based on the interviews conducted as post-experiment, the study speculates that two-step hypothesis by Itoh et al. (2017) may only apply to a subset of possessors of pitch class–color synesthesia. Among the 16 participants, four claimed that their color perception of pitch did not necessarily require pitch name recognition.

The study conducted a separate analysis of the data from participants who stated that their color perception was one-step during the interviews. The results demonstrated no significant difference in RTs between the color and pitch tasks under the solo conditions, which is inconsistent with the hypothesis of Itoh et al. (2017). This finding implies that the synesthetic cognitive mechanism of these participants does not align with the two-step process proposed by Itoh et al. (2017); instead, a possibility exists that it is one-step, as reported by the participants, which extends the hypothesis of Itoh et al. (2017). In fact, the reanalysis of the results based on the interviews indicates that the two-step group produced a result similar with the finding of Itoh et al. (2017). Although the existence of the one-step mechanism may be slightly subtle due to the small number of the participants (limited to only four participants), this aspect warrants an in-depth investigation in the future.

### 5.3 Selective applicability of rainbow-like theory to two-step synesthetes

The current study replicated the rainbow-like pattern proposed by Itoh et al. (2017). However, we found that if the reported colors of the two- and one-step groups are separately examined, the colors from the two-step group evidently align more closely with the rainbow-like pattern, while those reported by the one-step group seemingly diverge.

We reasonably speculate that this result may be due to different trigger mechanisms for synesthesia in the two- and one-step participants. Through the interviews, the study found that the synesthetic triggers reported by the participants could be divided into two categories, namely, one-to-one correspondence between sound and single tone and the generalization of other sensations. The majority of the participants who acquired synesthesia through the first method claimed two-step, whereas the majority of those who acquired synesthesia through the second method did one-step.

These notions may suggest that the manner in which synesthesia was formed could be the direct reason for the different mechanisms of synesthetes. To the best of our knowledge, this study is the first to report it. If this inference is applied to the 15 participants in Itoh et al. (2017), then we could speculate that the colors perceived as least rainbow-like by cases 3 and 10 may have been acquired synesthesia through the second method, namely, the one-step process (actually, in Itoh et al. (2017), cases 3 and case 10 self-reported that their synesthesia was one-step).

Therefore, why do two-step synesthetes exhibit a similar rainbow-like color perception? Based on the interviews, we reasonably conjecture that this aspect may be related to a number of common cultural imagery in Japan. During the interview, P12 noted that a favorite anime character during primary school may have partially influenced her color perception. In this anime, characters of different colors were given different names starting with pitch names. In addition, in a widely circulated Japanese-translated children’s song (the theme song of the movie Sound of Music), the lyrics mention that “*re* is the ‘le’ in ‘lemon”’ (Japanese does not distinguish between the l and r pronunciations), and “*so* is the ‘so’ in ‘sora”’ (sky), which may have led Japanese synesthetes to link the color of lemon yellow with *re* and the blue of the sky with *so*.

As for one-step synesthetes, their connections are relatively inconsistent, which may be due to the generalization from the impression of a particular song, which resulted in the decreased consistency of their color perception.

However, the question of whether or not synesthesia can, indeed, be acquired through postnatal training remains a research issue among researchers. A few studies contend that synesthesia is an innate capability (Bargary and Mitchell, 2008; Asher et al., 2009), while others argue that it can be acquired postnatally (Bor et al., 2014).

Undoubtedly, for participants with the two-step mechanism, their reported process of the acquisition of synesthesia seemingly lends more credence to the acquired perspective. Many of the two-step participants indicated that they developed synesthesia through methods akin to marking different musical notes with distinct colors on sheet music. In contrast, participants with the one-step mechanism frequently reported acquiring pitch class–color synesthesia by generalizing the image of previously heard songs to individual tones. However, many were unable to declare the origins of their initial color associations with such songs. This process appeared more implicit compared with the explicit learning described by the participants with the two-step mechanism. Whether or not one-step synesthetes acquire their capability through the postnatal implicit learning or possess an inherent propensity remains an intriguing avenue for future research.

### 5.4 The “training” of synesthesia

We posited earlier that two distinct mechanisms may be shaped by different methods of acquisition. Specifically, individuals who acquired pitch-class color synesthesia via one-to-one correspondence are more likely to exhibit a two-step mechanism, whereas those who acquire synesthesia through generalization tend to display a one-step mechanism.

However, whether synesthesia can be acquired postnatally through training remains a topic of ongoing debate among researchers. Some studies argue that synesthesia is an innate capability (Bargary and Mitchell, 2008; Asher et al., 2009), while others suggest that it can be acquired later in life (Bor et al., 2014).

Notably, for participants with the two-step mechanism, their reported experiences of acquiring synesthesia provide support for the acquired perspective. Many of these participants indicated that they developed synesthesia through explicit methods such as marking different musical notes with specific colors on sheet music.

In contrast, participants with the one-step mechanism often reported acquiring pitch-class color synesthesia by generalizing the imagery of previously heard songs to individual tones. However, many were unable to definitively explain the origins of their initial color associations with these songs. This process appeared more implicit compared to the explicit learning described by the two-step participants. Whether one-step synesthetes acquire their ability through postnatal implicit learning or possess an inherent predisposition remains an open question and a promising avenue for future research.

### 5.5 Pitch class–color synesthesia may facilitate the acquisition of AP beyond the critical period

As introduced in the Introduction, AP is considered an ability that is nearly impossible to acquire postcritical period. However, through the interviews, the study found that at least two participants (P4 and P8) claimed to have developed AP abilities after entering university. According to their self-reports, they both acquired synesthesia by chance during their early years, and although they did not systematically study music, undergoing structured musical training at university awakened their AP abilities. In the post-experiment interview, P8 mentioned that she believed synesthesia played a positive role in the acquisition of AP, as expressed in the following sentiment, “I felt that since I already had pitch class–color synesthesia, I should focus on acquiring absolute pitch.”

This aspect implies that pitch–color synesthesia and AP potentially share related mechanisms, and the formation of the former may promote the development of AP abilities, which may render acquiring it even beyond the critical period possible.

However, since the determination of AP ability in this study was solely based on self-reports and performance in pitch task (which included only a limited number of white keys), the participants’ AP abilities may have been overestimated. What P4 and P8 acquired might not be true AP but rather highly accurate Relative Pitch (RP), meaning their pitch recognition may still rely on a reference tone.

### 5.6 Synesthesia for microtones and chords

The synesthetic experiences addressed in this study specifically pertain to the color sensations of individual tones. However, during the interviews, several participants mentioned that they also perceive colors in relation to chords. The patterns of these color sensations widely varied among the participants. A few of them that their color perceptions for chords align with the color associated with the highest pitch within that chord. Conversely, others stated that their color perception for a chord entirely differed from colors associated with individual tones within that chord, which indicates that the resultant color may be a composite of the colors of each tone or bear no evident relation to the colors of individual tones. As such, the color perception of synesthetes in response to chords presents a compelling avenue for future research. The following questions emerge: what factors influence the different modes of color perception, and is there a close relationship with the manner in which synesthetes acquire their synesthetic abilities? Further studies are required to answer these questions.

## 6 Conclusion

### 6.1 Conclusions and significance

This study explored the mechanisms related to pitch class–color synesthesia by combining quantitative and qualitative analyses, thereby, providing a retest of the research conducted by Itoh et al. (2017).

The current results suggest that the conclusions drawn by Itoh et al. (2017) may pose certain limitations in the scope of application and that mechanisms of pitch class–color synesthesia that do not necessarily require pitch naming may exist. The difference in these mechanisms may be related to the circumstances under which synesthetes acquire synesthesia.

Furthermore, we identified two instances of individuals who claimed to have acquired AP beyond the critical period. Both of them acquired pitch class–color synesthesia at an early age. Although this study may potentially overestimate their AP ability, this finding could suggest that this form of synesthesia may share related mechanisms with AP and may promote the development of AP even beyond the constraints of the critical period.

Thus, the study infers that the achievements of this study has deepened the current understanding of pitch class–color synesthesia, which leads to the possibility of the acquisition of AP beyond the critical period and offers new avenues for the exploration of the relationship between synesthesia and AP.

### 6.2 Limitations and future directions

The tasks in our study differ from those in Itoh et al. (2017) in ways that may reduce their comparability. In Itoh et al. (2017), participants generated color qualia internally without visual prompts, whereas this study presented colors on-screen, potentially altering synesthetic processing. Furthermore, Itoh et al. (2017) utilized vocal responses, which are generally faster and yielded RTs below 1 second for color and pitch naming tasks. In contrast, the current study employed mouse and keyboard inputs, resulting in longer RTs and potential noise that may obscure the effects of the one- or two-step mechanisms. Additionally, this study used performance on the pitch task to evaluate AP ability. However, this task, which included only white-key pitches and had a limited pitch range, is likely to overestimate participants’ AP ability. This limitation particularly undermines the credibility of the claim that P4 and P8 developed AP after the critical period. Future researchers should conduct more rigorous AP ability tests before further exploring this topic.

Despite the qualitative analysis, which suggests the possibility of two- and one-step mechanisms and speculates on the reasons for these divergent mechanisms, this study lacks solid quantitative data to support these ideas. As such, further research is required to obtain data in the future. Similarly, the conjectures presented regarding the relationship between pitch class–color synesthesia and the formation of AP need to be followed up through future research.

Regarding the design of the experiment, although we tested the difficulty of the closed area judgment and silent reading tasks prior to the experiment and balanced task difficulty with the help of several graduate students from the university to which the authors belong, we may have underestimated the difficulty of the unfamiliar task of closed area judgment (see section 1.2 of Supplementary material details). During the experiment, a number of participants experienced difficulty in adapting to this task. Additionally, for a number of participants, the color options were more distinguishable than were the pitch name options, which made identifying corresponding color options when clicking with a mouse easy for them. This aspect resulted in shorter RTs for color tasks. Furthermore, in the dual tasks, the participants responded to both tasks, which may have led to bias due to the difficulty of multitasking. Future studies could instruct participants to respond only to auditory stimuli to eliminate this influence.

Finally, although the number of participants (*n* = 16) may not seem extremely small for a relatively rare condition such as synesthesia, future experiments may need to recruit as many participants as possible.

## Data Availability

The datasets presented in this study can be found in online repositories. The names of the repository/repositories and accession number(s) can be found in this article/Supplementary material.
