# Supplementary figures and images for: One- or two-step? New insights into two-step hypothesis and rainbow-like theory for pitch class–color synesthesia
Source: Front Psychol. 2025 Jan 10;15:1482714. doi: 10.3389/fpsyg.2024.1482714 (PMC11758358; doi:10.3389/fpsyg.2024.1482714)

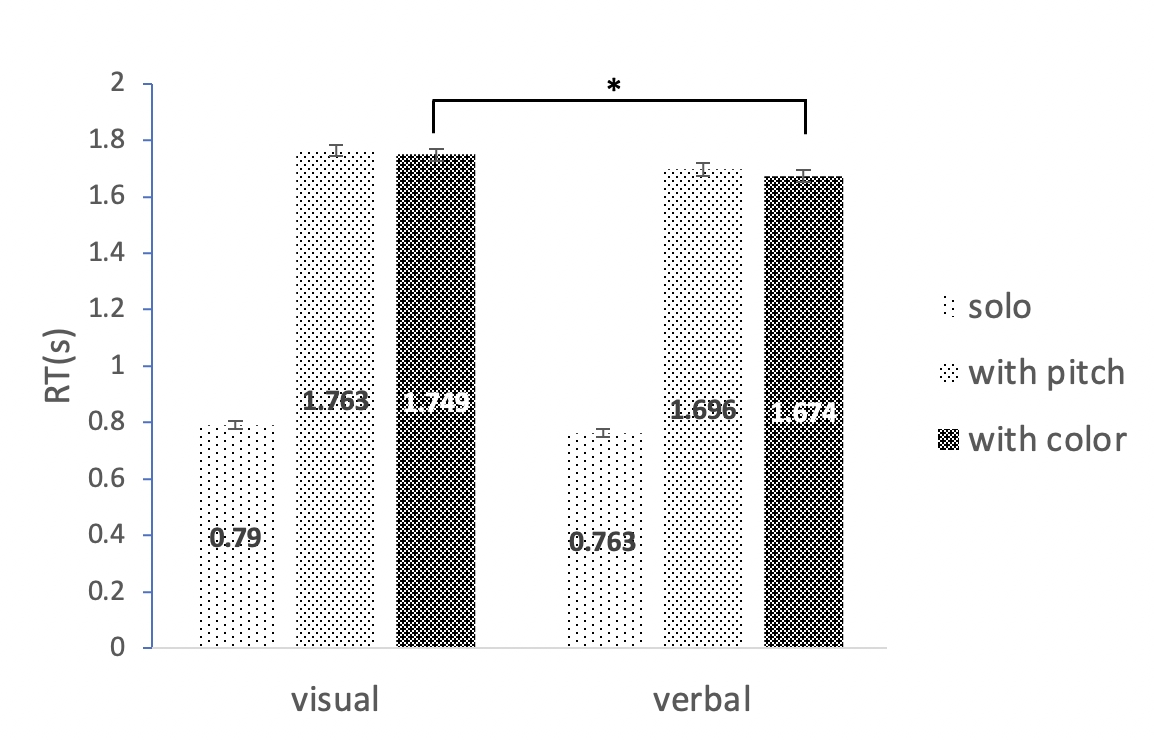

Supplement: Supplementary file 1 [file Supplementary_file_1.zip › Supplementary Figure 1(B).jpg]

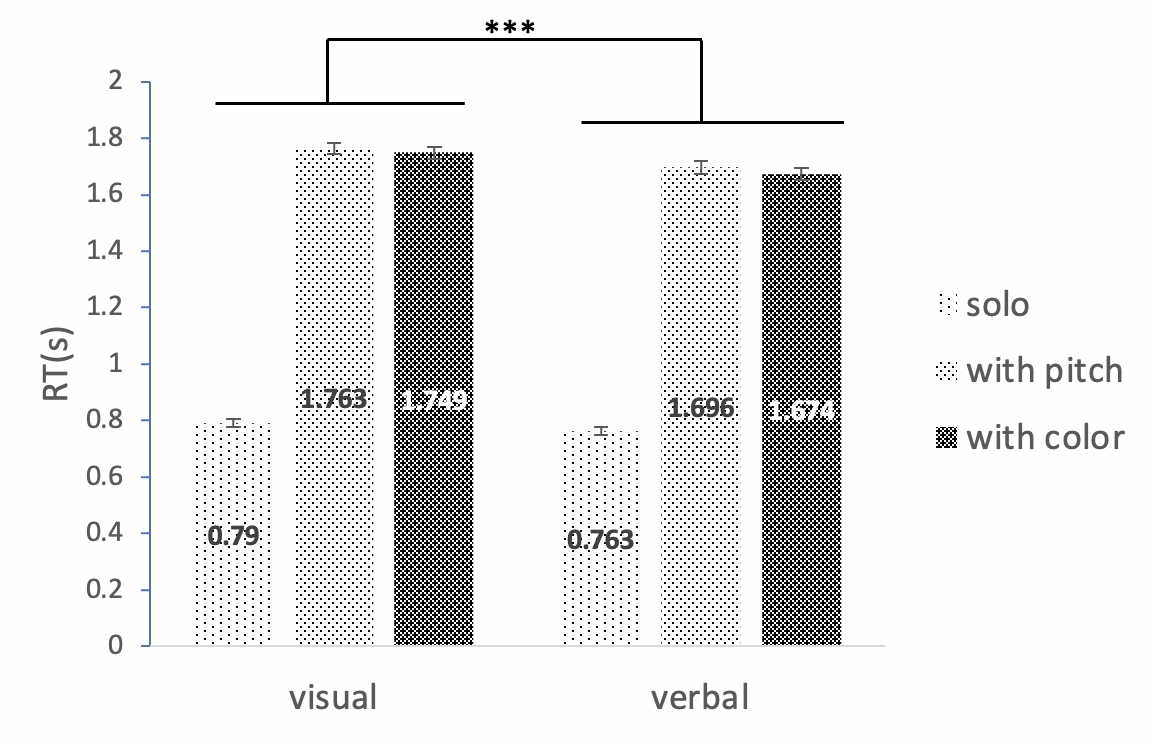

Supplement: Supplementary file 1 [file Supplementary_file_1.zip › Supplementary Figure 1(A).jpg]
