# Supplementary material for: One- or two-step? New insights into two-step hypothesis and rainbow-like theory for pitch class–color synesthesia
Source: Front Psychol. 2025 Jan 10;15:1482714. doi: 10.3389/fpsyg.2024.1482714 (PMC11758358; doi:10.3389/fpsyg.2024.1482714)
Supplement: Supplementary file 2 [file Data_Sheet_1.docx]

Supplementary Material

One- or Two-Step?
New Insights Into Two-Step Hypothesis and
Rainbow-Like Theory for Pitch Class–Color Synesthesia

Ang Cao^1^, Kazuhiro Ueda^1*^,

^1^Graduate School of Arts and Science, The University of Tokyo, Tokyo, Japan

*** Correspondence:**Corresponding Authors
[DiderD@outlook.com](mailto:DiderD@outlook.com) (Ang Cao)
[ueda@g.ecc.u-tokyo.ac.jp](mailto:ueda@g.ecc.u-tokyo.ac.jp) (Kazuhiro Ueda)

Keywords: pitch class–color synesthesia_1_, dual-task_2_, absolute pitch_3_, rainbow-like theory_4_.

# Supplementary Data

## The Number of Errors

The study recorded the number of errors committed by each participant across sessions. Supplementary Table 1 reports the results. The study observed that many participants committed more mistakes in the color tasks (total: 198) than they did in the pitch tasks (total: 41) and more mistakes in the visual tasks (total: 131) than they did in the verbal tasks (total: 73) regardless of condition. This trend may be due to the fact that the pitch and verbal tasks are more common in everyday contexts. Hence, these tasks were considered potentially more familiar and straightforward to the participants.

In addition, the study observed that the participants made the highest number of mistakes in the sessions in which the color and visual tasks was conducted. This notion may be due to the fact that the visual + color task was administered as the first dual task in the dual-task sessions for 8 out of the 16 participants despite the adoption of the current experiment of a Latin square balance of eight sessions.

## Difficulty Level of the Visual and Verbal Tasks

We employed a dual-task paradigm and designed interference tasks to test Itoh et al. (2017)’s two-step hypothesis. The rationale is as follows: the first step (the pitch name recognition process), should be more susceptible to interference from a verbal task that shares similar cognitive processes (see Lines 127–137 and H3 in main text for details).

This heightened susceptibility to interference is expected to result from the cognitive overlap with the verbal task, rather than the difficulty level of the interference task itself. Therefore, the visual and verbal interference tasks should not differ in difficulty.

### Pre-Test

Prior to the formal experiment, the study requested four graduate students, all of whom did not participate in the main experiment, to conduct the tasks to ensure equal levels of difficulty between the verbal and visual tasks. Analysis of the results revealed that the participants achieved consistent accuracy rates of 98.21% for the visual and verbal tasks. Additionally, according to the results of a *t*-test, no significant difference was observed in the average RTs between the two tasks (mean RT: the visual task = 0.8015 [*se* = 0.2812]; verbal task = 0.7528 [*se* = 0.1855]; *p* =.09). Therefore, no difference existed in the difficulty levels between the visual and verbal tasks.

### The RTs of the Visual and Verbal Tasks in formal experience

The study conducted a repeated-measure ANOVA to test this hypothesis. The independent variables were interference (visual/verbal) and response (none/pitch/color); the dependent variable was RT, as recorded during the visual and verbal tasks.

Inconsistent with the expectation, the main effect of interference was significant (*F* = 8.468, *p* =.004, *η*² =.020). RTs for the visual task were significantly longer than those for the verbal task (Supplementary Figure 1A). The result of post hoc comparison using Bonferroni’s correction, RTs for the visual task were significantly longer than those for the verbal task only when the response type was color (Supplementary Figure 1B).

These results may be due to the fact that the first dual-task administered on 8 out of the 16 participants in the dual-task sessions was the closed area judgment + color corresponding task, although the current experiment adopted a Latin square balance of eight sessions. In addition, the color and visual tasks were the ones that are not frequently witnessed in the daily life of the participants. This scenario may have resulted in longer RTs due to lack of practice and unfamiliarity with the task.

## Possible limitation caused by consistency test absence

We did not implement a rigorous consistency test as provided by Itoh et al. (2017), which may have introduced some errors into our study. In our experiment, we employed a two-step process to determine pitch-color associations: an online questionnaire followed by an in-person confirmation and modification session with the participants.

During participant recruitment, participants were asked to use an online color code picker to select the colors they perceived as corresponding to each pitch class and to provide the hexadecimal color codes for these selections. Once these color codes were collected, we displayed them on a monitor at the beginning of the offline session to allow participants to confirm whether the displayed pitch-color associations matched their perceptions. If they identified any discrepancies, they were asked to report the mismatched colors and modify them for use in the subsequent experiment.

Among all the participants, only P2 modified her color mapping during the offline session (specifically for do and fa), while the others confirmed that the colors displayed on the experiment computer were consistent with their perceived pitch-color associations. After completing the online questionnaire, participants scheduled the earliest available time to participate for the offline session based on their availability. Consequently, almost all participants completed the offline session within two weeks of filling out the online questionnaire—a notably shorter interval compared to the three-month gap reported by Itoh et al. (2017).

# Supplementary Figures and Table

**Supplementary Table 1** Number of errors per session per participant

| **Participant** | **Color** | **Pitch** | **Visual** | **Verbal** | **Color + Visual** | | **Color + Verbal** | | **Pitch + Visual** | | **Pitch + Verbal** | |
| --- | --- | --- | --- | --- | --- | --- | --- | --- | --- | --- | --- | --- |
|  |  |  |  |  | **Color** | **Visual** | **Color** | **Verbal** | **Pitch** | **Visual** | **Pitch** | **Verbal** |
| **P1** | 3 |  | 1 | 1 | 5 | 4 | 1 | 6 |  | 1 | 4 | 5 |
| **P2** |  |  | 3 |  | 1 | 12 | 1 | 1 | 1 | 4 |  | 1 |
| **P3** | 16 |  | 1 |  | 33 | 7 | 15 | 5 |  | 2 |  |  |
| **P4** |  |  |  |  | 1 | 2 | 5 | 3 | 2 | 1 |  |  |
| **P5** |  |  | 1 |  |  | 1 |  |  | 1 | 3 |  |  |
| **P6** |  |  | 1 |  | 2 | 3 | 2 | 2 |  | 3 | 2 | 1 |
| **P7** |  |  | 3 |  | 1 | 3 |  | 2 |  |  |  | 4 |
| **P8** | 8 | 4 | 3 | 2 | 2 | 3 | 3 | 4 | 5 | 4 | 10 | 8 |
| **P9** |  |  | 5 |  | 1 | 5 |  |  |  | 2 |  | 1 |
| **P10** |  |  | 2 | 1 | 2 | 2 | 2 |  |  |  |  | 1 |
| **P11** | 5 |  | 1 |  | 18 | 8 | 8 | 7 |  | 3 | 1 |  |
| **P12** | 1 |  |  |  |  | 1 |  |  |  | 1 | 1 |  |
| **P13** | 1 |  | 1 |  | 2 | 5 |  |  |  | 3 | 1 | 1 |
| **P14** | 2 | 3 | 9 | 4 | 2 | 5 | 2 | 5 | 1 | 3 | 3 | 4 |
| **P15** | 16 |  |  |  | 18 | 1 | 16 | 3 |  | 2 |  |  |
| **P16** | 1 | 1 | 1 |  | 1 | 2 | 1 | 1 | 1 | 3 |  |  |
| **Total** | **53** | **8** | **32** | **8** | **89** | **64** | **56** | **39** | **11** | **35** | **22** | **26** |

Note: The four columns following “No.” and “Participants” represent the number of errors for the four tasks under the solo conditions, and the next four columns represent the number of errors under the dual-task conditions.

**Supplementary Figure 1 (A)** RTs for the visual task were significantly longer than that for the verbal task. **(B)** Result of post hoc comparison using Bonferroni’s correction. Only when the response type was color, the RTs of visual task were significantly longer than those of the verbal task. In both panels, error bars indicate standard errors.
